# Ultrasound-Guided Percutaneous Antegrade Varicocele Embolization with Cyanoacrylate Glue as an Alternative to the Standard Retrograde Approach

**DOI:** 10.3390/diagnostics10060432

**Published:** 2020-06-25

**Authors:** Olivier Chevallier, Julie Pellegrinelli, Kevin Guillen, Romaric Loffroy

**Affiliations:** Department of Radiology, Section of Vascular and Image-Guided Therapy, François-Mitterrand University Hospital, 14 Rue Paul Gaffarel, BP 77908, 21079 Dijon, France; olivier.chevallier@chu-dijon.fr (O.C.); julie.pellegrinelli@gmail.com (J.P.); kguillen@hotmail.fr (K.G.)

**Keywords:** venous intervention, varicocele, testicular vein, embolization, cyanoacrylate glue

## Abstract

We report a case of a 29-year-old male referred to our hospital for endovascular treatment of a left-sided painful varicocele. Standard retrograde embolization via the left renal vein was not possible because of the presence of a left circum-aortic renal vein making the catheterization of the testicular vein not feasible. The patient was successfully treated via ultrasound-guided percutaneous antegrade access of the testicular vein at the inguinal level with subsequent cyanoacrylate glue embolization as a minimally invasive alternative to surgical therapy. This is a new approach to varicocele embolization when the left renal vein does not feed the varicocele.

**Figure 1:**
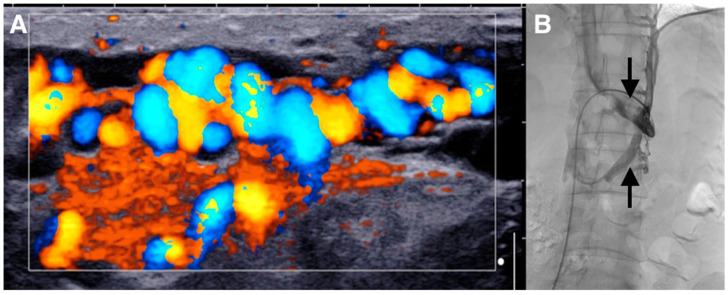
Varicocele repair is mainly indicated in young adult patients with clinical palpable varicocele and abnormal semen parameters or pain. Indeed, evidence from the literature demonstrates that varicocele treatment is associated with a significant improvement in sperm features and pregnancy rate compared to observation [1,2]. Many surgical techniques have been described for varicocele treatment. In recent decades, laparoscopic ligation, microsurgical varicocelectomy, percutaneous retrograde sclerotherapy or embolization, and antegrade scrotal sclerotherapy were the main alternatives to standard inguinal or suprainguinal surgical ligation. None of the previous techniques showed to be superior to the others in terms of success and complication rates. Only in recent years, microsurgical repair and sclero-embolization methods seem to be more effective than inguinal and suprainguinal ligation in terms of recurrence rate. Embolization has the advantage of being a minimally invasive procedure, performed under local anesthesia, with low cost and low risk. The most used approach is the retrograde endovascular approach, mainly with coils. However, nowadays an interventional radiologist’s preference is still the most relevant criteria to choose the approach and the embolic material for varicocele treatment [3,4]. In this case, we describe the use of glue by antegrade approach for varicocele embolization. We also describe our step-by-step technique. (**A**) The patient was a 29-year-old male who presented with a painful left-sided varicocele. Doppler ultrasound demonstrated a large left varicocele. Ultrasonographic criteria were usual for varicocele diagnosis: varicocele venous pampiniform plexus diameter > 3 mm and the retrograde flow into the plexus under Valsalva maneuver as shown here on color Doppler image [1]. (**B**) He was referred to interventional radiology for endovascular embolization. Standard retrograde embolization via the left renal vein by transfemoral approach was not possible, because there was a left circum-aortic renal vein making the visualization and catheterization of the testicular vein impossible (arrows).

**Figure 2:**
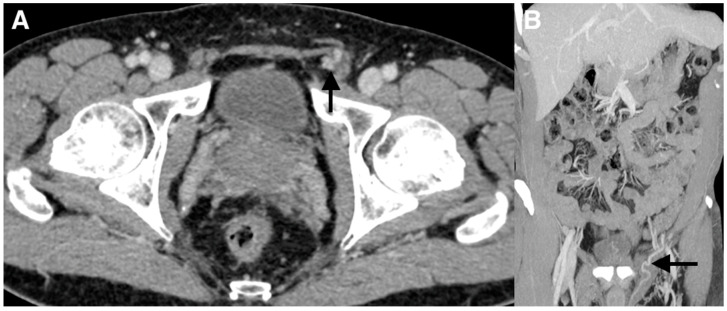
(**A**,**B**) Subsequently, a contrast-enhanced computed tomography (CT) imaging study with frontal reconstructions was obtained to evaluate the vasculature. The CT scan did not demonstrate a left testicular vein arising from the left renal vein. The left testicular vein at the level of the inguinal canal was visualized feeding the left-sided varicocele (arrows).

**Figure 3:**
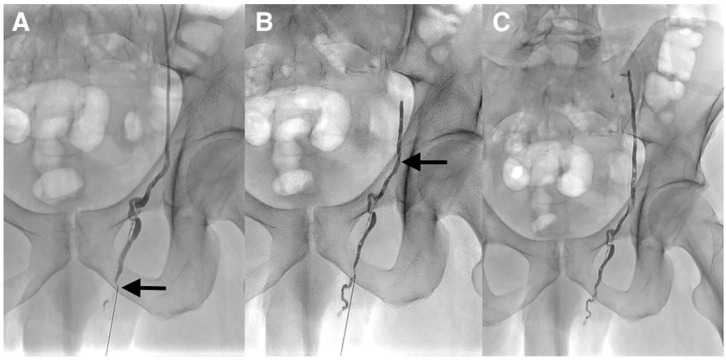
(**A**) The lack of visualization of the left testicular vein was felt to be due to its possible small caliber; therefore, the patient was scheduled for the antegrade embolization of the left testicular vein. The procedure was performed under local anesthesia. The testicular vein was percutaneously punctured with a 21-gauge metallic needle under ultrasound guidance at the left inguino-scrotal level by direct antegrade approach. A venogram through the needle was performed and demonstrated that the varicocele was supplied by the normal testicular vein (arrow). It was impossible to identify drainage into the left renal at the upper part on the venogram because of the risk of malposition of the needle during contrast injection. One explanation for the failure of the retrograde embolization was the competence of the first testicular vein valve at the level of the renal vein as often described despite the dilation of the testicular vein and the varicocele below. This valve could not be crossed. (**B**,**C**) Because of the inability to catheterize deeper the testicular vein with a microcatheter, cyanoacrylate glue embolization was directly performed through the 21-gauge needle. A mixture of Glubran^®^2 (GEM, Viareggio, Italy) and Lipiodol^®^ (Guerbet, Aulnay-sous-Bois, France) in a 1:1 ratio was gently injected until the occlusion of the two-thirds of the testicular vein above the puncture site and few centimeters below this point (arrow). The catheter was removed and hemostasis was obtained with manual compression. The patient tolerated the procedure and was discharged the same day. He returned to his normal activity without pain and was asymptomatic at the 6 months follow-up. Varicoceles are often mainly treated with percutaneous embolization using fibered coils or sclerotherapy [1]. Coils have the advantage of being easy to use, especially detachable coils. The main drawbacks are the risk of migration if the size is not appropriate and the risk of recanalization, since small collaterals cannot be accurately treated with them. In addition, many coils may be necessary. The limitations of sclerotherapy are: the risk of migration/stroke, allergy, unpredictable diffusion space, total amount limited, inefficient in the case of large varicocele if used alone. The main advantage of sclerotherapy is the very low cost. Liquid embolic agents such as cyanoacrylates are less popular despite many advantages such as lower cost, faster procedure, and a higher capacity of the vein filling, especially collaterals [2]. A deep learning curve is necessary and the choice of low dilution with lipiodol is mandatory to avoid migration in the case of reflux. The entrapment of the microcatheter is exceptional in clinical practice if embolization is performed properly. There are not only a few cases with percutaneous antegrade varicocele embolization via the testicular vein at the level of the pubis, but also no reports of cyanoacrylate glue embolization in such a setting as we herein described [3,4].
